# Effect of Television on Obesity and Excess of Weight and Consequences of Health

**DOI:** 10.3390/ijerph120809408

**Published:** 2015-08-12

**Authors:** Anna Rosiek, Natalia Frąckowiak Maciejewska, Krzysztof Leksowski, Aleksandra Rosiek-Kryszewska, Łukasz Leksowski

**Affiliations:** 1 Department of Public Health, Faculty of Health Sciences, Nicolaus Copernicus University in Toruń, Bydgoszcz 85-830 & Ross-Medica, Poland; 2 Department of Public Health, Faculty of Health Sciences, Nicolaus Copernicus University in Toruń, Bydgoszcz 85-830, Poland; E-Mail: natalia.framac@vp.pl; 3 Chair of Public Health Department, Faculty of Health Sciences, Nicolaus Copernicus University in Toruń, Bydgoszcz 85-830 & Department of General Thoracic and Vascular Surgery, Military Clinical Hospital in Bydgoszcz, Poland; E-Mail: leksowski@poczta.onet.pl; 4 Department of Inorganic and Analitycal Chemistry, Faculty of Pharmacy, Nicolaus Copernicus University in Toruń, Bydgoszcz 85-089, Poland; E-Mail: ola.chemia@wp.pl; 5 Department of Rehabilitation, Faculty of Health Sciences, Nicolaus Copernicus in Toruń, Bydgoszcz 85-094, Poland; E-Mail: leksowski.lukasz@wp.pl

**Keywords:** public health, human diseases, nutrition, risk factors, well-being, obesity, TV watching and health

## Abstract

The epidemic nature of obesity in industrialized countries is a serious health and social concern. The number of obese people has significantly increased in the past 20 years. In Poland excess weight and obesity are a serious epidemiological concern. In terms of the number of overweight people, Poland is a leader in Europe. Therefore, indicating many serious health concerns that are the natural consequences of this phenomenon has become important from the point of view of public health. This work identifies numerous diseases which are a direct consequence of obesity due to bad eating habits and lack of physical exercise among Poles. It discusses the negative effect of television and food commercials contributing to an increase in obesity, not only among adults but also among children. This is an overview forming grounds for further studies into ways of preventing the development of diseases due to obesity, both in Poland and in the world.

## 1. Introduction

According to figures published by the Food and Nutrition Institute in Warsaw the prevalence of overweight (including obese) children in European developing countries after 2000 exceeded 20%, while in the US the figure is as high as 35% [1]. The study “The health status of the Polish population” conducted by CSO in 2009 showed that 61.4% of men weigh too much, and this results in one of the highest values in the EU countries [1,2]. Statistical analysis indicates that, in Poland, the prevalence of overweight and obesity in the age bracket from 1 to 18 ranges from 14.5% in girls to 19.9% in boys [1]. The study shows that in Poland the most common cause of obesity are poor eating habits and low physical activity.

Improper health behaviors often prevail in the whole family; obesity is observed in both parents and children. However, in the case of children and adolescents is a grows exponentially. Children aged 2–14 spend an average of 2.4 h a day in front of a TV screen or a computer monitor [1,2]. In Poland the group, which allocates five or more hours a day to TV screens or computer monitors, is 6.9% of all children [2]. Children are no longer interested in outdoor activities. Young people spend a lot of time in front of their computers, playing games and browsing the Internet, but this does not involve physical effort; hence, there is no energy loss. As a result, more and more often there is the problem of overweight and obese children and adolescents, which persists into adulthood. Currently, the excessive weight has been observed in every other adult Pole and most problems with excess body weight are just among men [1,2].

Given that lifestyle and eating habits acquired at a young age translated into health in adulthood, making a healthy lifestyle a subject matter of interest of public health care institutions has become an important issue so that we can enjoy good health into old age.

## 2. Prevalence of Obesity

About 20% of the world’s population is obese; thus, obesity is classified among the diseases of civilization. The epidemic nature of obesity in industrialized countries is a serious health and social concern. The number of obese people has significantly increased in the past 20 years. In certain developed countries, 50% to 65% of the total population are overweight or obese, which means that only 1/3 of the people have normal body weight. 

Obesity is the sixth most important risk factor in terms of the number of deaths in the world. In 1997 the WHO officially declared obesity to be a chronic condition which requires treatment, fosters the development of other diseases, and is connected with increased mortality. 

The UK Department of Health forecasts that if an upward trend in obesity is maintained, by 2050 the average length of life of males will be reduced by five years as a result. American researchers have found that by 2030 as many as 78.9% of adult Americans will be overweight or obese. If the epidemic is not contained, the present population of Americans will live shorter than their parents. 

According to the WHO in the European Region the number of obese people is three times higher than 20 years ago. In Poland, excess weight and obesity are a serious epidemiological concern and, in terms of the number of overweight people, Poland is a leader in Europe [3]. 

During the past 30 years, in most countries of the world a significant increase in the rate of incidence of overweight and obesity in the developing population has been observed. Based on research carried out in different regions of the world, it is estimated that the number of obese people aged up to 18 tripled in the 1990s [4]. 

Similar to other countries, in Poland an upward trend has been observed in the incidence of overweight and obesity in the developing population and the number of reports on overweight and obesity among children and young people is spiraling upwards. According to studies carried out by the Food and Nutrition Institute under the National Programme for Prevention and Treatment of Obesity, the problem of overweight and obesity refers to about 12%–14% of children and is different in respective regions [5].

## 3. Factors Associated with Increases in Obesity

Obesity is a gain in body weight, conditioned by the accumulation of excess body fat, significantly above the norms set for specific ages, races and sexes, and exceeding the physiological needs and adaptability of the human body [6]. The appropriate body mass is determined by the BMI (Body Mass Index) [7] expressing the weight to height ratio. A BMI greater than 30 kg/m^2^ means obesity. With BMI exceeding 40 kg/m^2^ obesity is classified as very severe and there is a risk of negative health outcomes [8]. For definitions of obesity and overweight in this manuscript, authors accepted the International Obesity Task Force (IOTF) criteria, developed in 2005 by a group of IOTF experts, who extrapolated the adult BMI cutoff points for overweight (25 kg/m^2^) and obesity (30 kg/m^2^) [9]. Typically, the prevalence of overweight and obesity is estimated by categorizing individuals according to their body mass index (BMI) (kg/m^2^); the accuracy of this approach relies on the relation of this measure to the percentage of body fat [9]. The use of BMI is a widely accepted and an affordable method to infer body composition in children and adults [9], often used to diagnose overweight and obesity [10,11]. 

There are many reasons for being obese. Heritability *et al*. [12], hormonal disorders, nervous system factors, *i.e*., satiety and appetite disorders [13], genetic factors, including genetically determined syndromes, and hypothalamic damage [14] are the causes of obesity which cannot be controlled by the affected individual. However, the most frequent causes of obesity, given the variety of everyday conveniences, include: overeating, lack of physical activity, as well as mental and behavioral conditions [12]. 

At present, obesity is considered a chronic disease which must be treated like any other medical condition, and if not treated it leads, insidiously, to the development of numerous diseases. It has an epidemic-like nature and is not only one of the main causes of morbidity and mortality, in particular in developed countries, but also causes huge social and economic burdens [15]. In addition, obesity affects the functioning of many organs. Although the share of obesity in the pathogenic chain of diseases of many organs has not been clearly proven, there is no doubt that it fosters some of them and contributes to their aggravation and increases the incidence of complications.

Although obesity is widespread and continues to be a leading public health problem in the U.S. [16,17,18] the problem of obesity is also a huge problem in European societies. The main reason for that are the changes in lifestyle. Both in U.S. and in European countries (also in Poland) obesity is primarily a result of individual behaviors and environmental factors that lead to excess caloric intake and inadequate amounts of physical activity [19,20] (USDHHS, 2001; USDHHS, 2003). Examples of such individual behaviors and environmental factors are:

Increased snacking [1,2,21,22,23],
Larger portion sizes [1,2,24,25,26],
Higher calorie-density of foods [1,2,27],
Inadequate amounts of physical activity and increased media use (such as: watching television, and viewing computer screens) [1,2,28,29],
Mass production of food (fast food) using cheap ingredients e.g., corn syrup instead of sugar which has more calories than sugar [1,30,31].

All of these factors are the effects of globalization and affect, at least to some extent, obesity in our society. For this reason, the fight against obesity has become a global problem in many countries. It also occupies a special place in public health due to numerous health complications and diseases caused by obesity.

## 4. Effect of Mass Media and Lack of Physical Activity on the Incidence of Obesity

Commercials and mass media have a significant effect on our eating habits. The amount of time spent in front of the television and the content watched can be a reason for developing obesity. Television not only contributes to physical inactivity but commercials and other programs also encourage us to eat more. TV viewing (TV mobile screen watching) is a contributing factor to childhood obesity. It takes away from the time children spend on physical activities and also leads to increased energy intake through snacking and eating meals in front of the TV. Those habits of “sit time” and “snacks”, and also product advertisements on TV, influence children to make unhealthy food choices [32,33]. The results of studies indicate that an overt or covert food theme is omnipresent on television [34,35,36,37]. Usually high-calorie meals and snacks of little nutritional value, low in protein, vitamins or minerals are shown. It was also determined that, on average, references to food on television are made ten times an hour, excluding commercials [34,35,36,38]. Many studies regarding a positive correlation between the time of watching television, number of sweet drinks, and risk of developing obesity were also carried out in Poland. A study in the journal Appetite found that the “effects of television watching on food intake extend beyond the time of television watching to affect subsequent consumption” [39]. This is also confirmed by researchers in Poland [2,40] and by the Polish Central Statistical Office [1]. In other words, watching TV and time spent in front of the screen are clearly associated with unhealthy dietary behaviors in children, adolescents, and adults [2,40,41]. People who watch television more rarely, and drink less sweet drinks during the day, were characterized by lower BMI [2,35,37,40]. In 2003 the World Health Organization (WHO) and the Food and Agriculture Organization of the United Nations Organisation (FAO) officially reported that commercials of food products directed to children can foster the development of obesity and, in Poland [37,42,43], TV commercials addressed to schoolchildren and youth are dominated by high-calorie products. 

Many studies regard commercials as a causative factor supporting the spread of the epidemic of obesity among children and young people [1,2,32,35,36,39,40,41,42,43,44]. An average child watches television for four hours a day [35]. Every additional hour of watching TV per week increases the risk of developing obesity in school children by three per cent [37]. 

Although Sigman in his study [45] suggests that health risks are reported to occur beyond exposure of two hours of watching TV per day, the average child is exposed to three times this amount. A robust initiative to encourage a reduction of daily TV watching could lead to significant improvements in child health and development. A study of 5–6-year olds found that both active TV viewing and background “passive” TV exposure was related to shorter sleep duration, sleeping disorders, and overall sleep disturbances. Moreover, passive exposure to TV of more than three hours a day was strongly related to sleep disturbances’ therefore, “parents should control the quantity of TV viewing and  limit children’s exposure to passive TV watching as well” [46,47].

Recent studies have revealed that obese children watching commercials eat twice as many low-value products (such as sweets, chips, fast food, and products with a low nutritional value and little vitamins) and the most obese ones choose the least healthy products. 95% of advertising materials displayed in US schools promote fast food, sweets, and sweet drinks despite the negative opinions of numerous consumer organizations and American pediatric associations [48]. Such negative trends are similarly intense in the European Union [49] and in Poland [1,2,40], although—recently, in particular—many regulations have been introduced which require member states to provide young consumers with special protection [2,40]. Experiences from Poland show that a high percentage of obese/overweight children and insufficient knowledge of nutrition may consequently result in increased risk of cardio-vascular diseases in the adult population [40]. For this reason, educational programs for children and young people have been introduced in schools. They detail the need for consumption of vegetables, fruits, and vitamins in daily diet. The Polish government also introduced a list of products banned for sale in schools, but even these actions are not enough to effectively counteract obesity [2,40]. 

The study findings suggest [50,51] that parents choosing to keep their children active and away from TV or computer screens, even for a short time daily, may see a small improvement, more activity and less sedentariness, in the after-school period. In the aspect of obesity, more recent qualitative research suggests that it is also an important time to reduce children’s daily screen viewing time [50,51]. Even such a small change as approximately 5 min more of activity and 5 min less of sedentary time leads to a reduction of obesity and diseases caused by them, both in adults and children. The parent of obese children may find it difficult to make such a change and enforce it on their children, however, it will not only be beneficiary for the kids themselves, but also for the national healthcare systems (less money spent on treating obesity-related diseases). Although time spent viewing television appears to be stable [51,52], leisure time exposure to console-based electronic games and computing is increasing rapidly [51,52], as is the increase in mobile smartphones and touch screen tablets that are used for electronic gaming, social networking, video viewing, and Internet browsing [51,53,54]. The time spent by young people on screen viewing is increasing and contributes to the development of civilization diseases. Given this high sedentary exposure, healthcare practitioners should use all available opportunities to encourage children (and their parents) to be more active and less sedentary [51]. Although children are more susceptible to lesions due to prolonged television viewing, the problem of obesity, and its complications, the disease also affects adults. 

Wijndaele *et al*. [55] recently reported that a one hour per day increase in television viewing was associated with a 6% increased hazard for total fatal or non-fatal cardiovascular diseases, and an 8% increased hazard for coronary heart disease and early death increased independent of gender, age, education, smoking, alcohol, medication, or diabetes status [45,46,55,56]. Other researchers also emphasize that sedentary behaviors, especially TV watching, were associated with significantly elevated risk of obesity and type-II diabetes [57] and, compared with other sedentary activities such as reading or driving a car, TV watching contributes to reduced metabolism [44,57,58]. 

Excessive TV viewing is associated with many disease risks and is associated with numerous risk factors, including poor diet, lower socio economic status, obesity, smoking or depressive symptoms [1,2,40]. There is emerging evidence that too much TV watching also increases the risk of weight-related chronic diseases in Polish society [1,2,40]. This fact is also confirmed by the Nurses’ Health Study that followed more than 50,000 middle-age women for six years. For every two hours the women spent watching television each day, they had a 23 percent higher risk of becoming obese and a 14 percent higher risk of developing diabetes [57]. In Poland we observe the same tendency, but the target group which spends more time watching television and have less physical activity are men [2,40]. For this reason, men, more often than women, in Poland succumb to cardiovascular disease, type-II diabetes, and depressive symptoms, caused by excess weight. As a result, the Polish population statistics show that men live shorter by 10 years than women. As we can see, mass media may cause overweight and obesity in adult men, children, and adolescents. For this reason, preventive programs to combat obesity in Polish society are directed mainly to those target groups.

In conclusion, all illustrated data provide strong evidence that sedentary behaviors, especially prolonged TV watching, are directly related to obesity, diabetes risk, heart diseases, and even increased risk of early death. 

Although there is strong evidence showing the need to increase physical activity not only in Polish society, in order to improve health, the belief of the population without the cooperation of the media is relatively hard to enforce and the activities undertaken by the Polish state in this respect are ineffective. Globalization and the omnipresence of the media exert growing influences on public health and the health of individuals, e.g., some television activities which support health-promoting trends, virtually unnoticeable to the average viewer, are referred to as telepreventive medicine [59]. Although at this point the television is put in a bad light, especially as it impact negatively on the health of the population, the authors recognize the large role of national governments in changing this situation. Close cooperation of the government and the health sector with the media to introduce regulations on advertising only healthy products dedicated to children (such as vegetables and fruits), as well as the implementation of compulsory education for both nutrition and promoting physical activity in society, can stop the advancing process of the growth of obesity in Europe. Moreover, such regulations, if implemented, would likely be effective—and cost effective—for the public health sector.

## 5. Physical and Psychological Consequences of Obesity

Apart from adversely affecting one’s physical condition and self-esteem, obesity may lead to a number of serious diseases.

## 6. Diseases Caused by Obesity

### 6.1. Cardiovascular Diseases

The influence of obesity on the incidence of cardiovascular diseases is indisputable [60]. The incidence, intensity, and as a consequence, the effect on the quality of life and increased risk of early death as a result of cardiovascular complications related to obesity are determined by the degree, duration and pathogenic characteristics of obesity [61]. Obesity fosters the development of hypertension, vascular atherosclerosis (including coronary atherosclerosis), varicose veins, and venous thrombotic events (cerebral strokes, myocardial infarction) [60]. 

The blood of obese people has a different viscosity and flow rate through the capillaries [60,62,63]. The blood flow in an obese person does not exceed 60% of the blood flow characteristics of a person with normal body weight [15,60]. Also, the response of capillary vessels to constricting stimuli is attenuated [60,62,63]. The resistance of the walls of capillary vessels to negative pressure is decreased and they break [15,60,62,63,64]. Obese people tend to develop subcutaneous haematomas, which is a consequence of an increased permeability of capillary walls.

The increased mass of body fat and its active metabolism requires intense blood flow. Obesity increases the rest flow of blood through body fat many times. The system adapts to this condition by increasing the circulating blood volume, stroke volume, and cardiac output, and the heart responds with hypertrophy. Obesity also causes morphological changes of the heart by increasing its adiposity as well as fat infiltration within the cardiac muscle and its fatty degeneration. A specific obesity cardiomyopathy develops, which initially impairs the systolic, and later also diastolic, activity of the heart, leading to the development of cardiac failure [15,64]. The disease often affects the left ventricle; the hypertrophy is usually asymmetrical and it affects the interventricular septum. Ventricular volume is normal or reduced, and hypertrophy is often more significant in this area [65].

In obesity the amount of body fat is increased both in the pericardial area, in particular within the right ventricle and near the apex, and in the muscle tissue of the heart. Numerous cells containing fat may appear between cardiac muscle fibers and the amount of fat in the myocytes increases. This is a multiple increase compared to the normal amount of fat in the cardiac muscle. Apart from the amount of fat, the hypertrophy of the cardiac muscle is significant in terms of the heart weight gain in obese people. The hypertrophy is generally proportional to absolute body weight and the area it covers. Increased heart rate, also during rest, must also be considered a reason for cardiac hypertrophy in obesity. The heart rate of an obese person is on average 50% faster than that of a person with normal body weight [61]. Increased heart volume and the degree of cardiac hypertrophy in obese people do not catch up with the increase in the overall body weight. Thus, symptoms of cardiac failure occur. 

Obesity is also a factor increasing the risk of ischemic heart disease. It was demonstrated that obesity, and in particular central obesity, causes numerous interrelated metabolic, inflammatory, oxidative, hemodynamic, and thrombotic disorders which damage the vascular wall, impair regulatory reactions of the endothelium, and foster the build-up of atherosclerotic plaques and clots [61,66]. Atherosclerotic lesions pathomorphically quite typical of obesity occur in the coronary arteries, and at the same time in the carotid and cerebral arteries, the main artery, the aorta, and the peripheral arteries of the lower extremities. Among other things, overweight and central (abdominal) obesity stimulate the emergence of many strong risk factors related to atherosclerotic coronary disease [61,66].

Unhealthy lifestyle, fatty food, irregular meal times, and in consequence obesity, considerably contribute to the incidence of atherosclerosis, which, due to its manifestations, such as coronary disease and stroke, is the most frequent cause of death globally [67,68].

### 6.2. Respiratory Disorders

Obesity can lead to various respiratory disorders. It has two mechanical effects on the respiratory tract. As a result of excess soft tissue, the chest becomes more rigid or less flexible, thus more effort is required to expand it. Massive accumulation of soft tissue in the abdomen increases the pressure on the abdominal organs, forcing a higher rest position. The rigid chest wall reduces tidal volume and increases dead space ventilation [69]. This causes hypercapnia; that is, the partial pressure of carbon dioxide in blood elevated above the norm, which is 45 mmHg. The high rest position of the diaphragm in obese people, as a result of increased abdominal pressure, is connected with reduced dilatation of the lungs and obstruction of the small airways and the pulmonary alveoli in the lung base. Thus, certain areas are less ventilated. 

Many obese people suffer from upper airway collapse in obstructive sleep apnoea [69]. This is a respiratory disorder occurring during sleep characterized by multiple pauses in breathing or by considerably reduced flow of air through the airways at the throat level—abundant fat cover in the area of the neck reducing the throat passage. Along with increasing obesity the disease becomes more critical [70]. Most obese patients show arterial blood gas disorders, which is a consequence of the high position of the diaphragm, obstruction of the airways and the pulmonary alveoli, and irregularities in the ventilation-to-perfusion ratio. If the partial pressure of carbon dioxide in arterial blood is elevated, the term “obesity hypoventilation syndrome”, also known as the Pickwickian syndrome, is used [69]. In this condition, obesity impairs pulmonary ventilation and affects various respiratory components, e.g., leptin resistance. In patients suffering from obesity hypoventilation elevated concentrations of leptin are observed. The resistance of leptin receptors is one of the suggested mechanisms accounting for the lack of compensatory growth in minute ventilation (leptin increases minute ventilation) [71]. 

Environmental factors such as changes in eating habits, and in particular an increasing number of overweight and obese people, are also deemed to be the reasons for the increased incidence of bronchial asthma. Most studies have revealed that obesity increases the risk of asthma. Many researchers use the index of central obesity, claiming that it shows a large correlation with the occurrence of asthma and its severity [72]. Numerous cross-sectional studies indicate that asthma occurs more frequently in people with a higher BMI. In comparison with normal body weight, overweight increases the risk of asthma by 38%, while obesity by 92%, which is similar for both women and men. Also, the symptoms of asthma are more intense in obese people [73,74]. Such patients need more anti-asthma drugs and require emergency aid more often than slim people. In obese people asthma is the most severe. In the case of patients with higher BMI the control of asthma is worse and it is difficult to change from a lack of, or partial, control into full control of the disease, which can be related to the concomitance of diseases often accompanying obesity, e.g., sleep apnea [72].

### 6.3. Diabetes

Type-II diabetes is also associated with obesity. Diabetes is a term denoting a large group of diseases and metabolic disorders with separate etiology characterized by pathological hyperglycemia. The morbidity rate related to type-II diabetes is proportional to the average BMI characteristic of specific populations. In people with a BMI above 30 kg/m^2^ the rate of morbidity is about seven times higher than in people with normal BMI, *i.e*., below 25 kg/m^2^ [75]. In the treatment of type-II diabetes associated with obesity the first recommended activity is reducing the body weight to normal level, and then introducing muscle training, healthy life style and, only then, pharmacological treatment. It is also important to maintain basic and reactive secretion of insulin in response to a meal or glucose. The risk of developing type-II diabetes in people with impaired glucose tolerance is decreased by a BMI-reducing diet. If the BMI is reduced by more than 10 % the metabolic control of type-II diabetes is facilitated [76,77]. 

**Figure 1:**
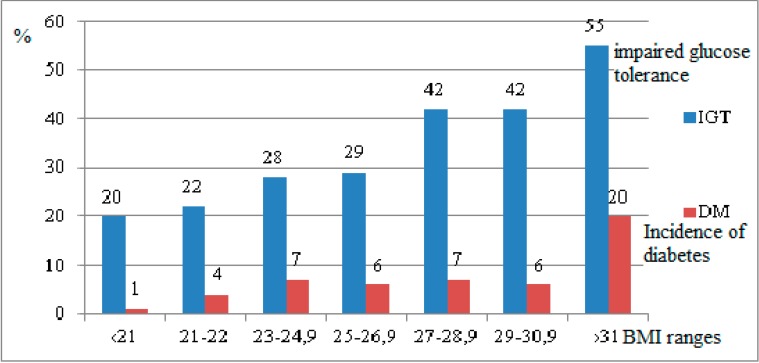
The likelihood of developing diabetes increases along with the degree of obesity, IGT-impaired glucose tolerance, x axis—BMI ranges, y axis—Incidence of diabetes and IGT (%) [79].

Diabetes develops considerably more frequently in overweight and obese people and the incidence of diabetes increases along with the degree of obesity [78]. Figure 1 also illustrates an increase in the incidence of carbohydrate intolerance along with an increase in BMI [79].

In about 50%–60% patients with type-II diabetes a concomitant condition is arterial hypertension. It is often associated with central obesity, manifested as a metabolic syndrome [75]. The causative factors of the metabolic syndrome include central obesity and insulin resistance. The untreated syndrome leads to manifest type-II diabetes and to premature development of atherosclerosis and their complications. Excess abdominal fat is evidenced by an increased waist circumference (≥80 cm for women and ≥94 cm for men). An indication can also be a case, without obesity or being overweight, when it is suspected that only the excess intestinal fat is responsible for insulin resistance [80].

Obesity predisposes to gallstones, gastroesophageal reflux disease and fatty degeneration of the liver [81]. 

Most frequently in such a case we deal with non-alcoholic fatty liver inflammation. It has a high rate of incidence and develops in 70% of obese people, with diabetes and hyperlipidemia. This condition is manifested by fatty degeneration of the liver and inflammatory infiltration of the lobules and portals. In 20%–40% of the cases of this disease liver fibrosis and cirrhosis develop [82]. For treatment purposes a reduction in body weight is recommended, along with the treatment of hyperlipidemia and diabetes. 

Similarly, the gastro esophageal reflux disease develops in obese smokers aged from 30 to 60 who have recently gained weight [83]. 

### 6.4. Kidney and Cancer Diseases

Obesity also leads to renal failure resembling focal segmental glomerulosclerosis (FSGS). In contrast to its idiopathic form, FSGS related to obesity is characterized by less intense clinical symptoms and slower progress until the end-stage renal disease develops. 

Obesity can also cause diseases affecting the glomeruli of the nephron; that is, glomerulopathy, where weight loss is of fundamental importance to successful treatment. Obese people also have proteinuria. It was found that the higher the BMI, the higher the risk of chronic renal disease even in people who previously had normal body weight. Excessive weight can also intensify the progress of chronic diseases of the kidneys. It was demonstrated that in patients suffering from IgA nephropathy being overweight contributes to faster loss of the renal excretory function.

In obese patients found eligible for a kidney transplant the activation of the transplanted organ is observed to be significantly more frequently delayed or the graft prematurely loses its function (a type of physiological reaction in the body of the organ recipient under the influence of lymphocytes with foreign antigens) [84].

Obese women more often develop gallbladder cancer, endometrial cancer, and breast cancer. Obese people of both sexes more often develop colorectal cancer but this may also be connected with a wrong diet or lack of physical activity [85]. Obesity is also a factor increasing the risk of esophageal adenocarcinoma [86].

### 6.5. Joints and Bones Deformity and Skin Lesions

Obesity and being overweight can lead to damage of locomotor structures and functions as a result of mechanical overload and direct effect of obesity pathogenesis, *i.e*., increased adrenergic tension, certain hormones, and inflammatory cytokines and metabolic influences. These mechanisms lead to locomotor diseases, usually of a degenerative nature. Obesity tends to be a cause of *pes planus* and *pes valgus* or knee deformity. 10% of people whose weight corresponds to 125% of normal weight and about 40% with a higher degree of obesity have *pes planus* (flat foot). Adult and elderly obese people also show signs of bone reconstruction and symptoms of osteoporosis [87,88]. Musculoskeletal pain is a particularly significant clinical symptom of loco-motor disorders. 

Osteoporosis of ankle and knee joints is about 4–8 times more frequent in people with a BMI > 30 kg/m^2^ compared to people with a BMI < 25 kg/m^2^ [87,88]. It occurs more often in women than in men and, in particular, is characteristic for people whose obesity developed at an early stage of their lives. Normally obesity also causes bilateral arthrosis of the hip joints, that is a risk factor leading to femoral fractures. The low back pain syndrome develops in children and young people but the morbidity rate is proportional to age and to the BMI [15].

Given the increasing number of overweight and obese people and the aging of society we can expect an increase in the number of people with excess body weight undergoing operative treatment of degenerative disease of the knee joints. It is also feared that obese people following knee joint endoprosthesis implantation, as a result of increased pressure on the elements of the endoprosthesis, can develop complications more often. It is also noteworthy that obesity reduces the mobility of the endoprosthesis of the knee joint and increases pain in the joint after surgery [89].

Obesity is connected with the presence of various skin lesions [90,91]. They can be a consequence of metabolic disorders, maceration, and occlusion or impeded or sometimes impossible skin care. They lead to impairment of skin sensation, temperature control, foot deformation, and vascular failure [90,91,92], acanthosis nigricans, hyperandrogenism (causing hair loss), and hirsutism (excessive male-pattern hair growth in women). Mechanical expansion of the skin results in stretch marks. Obesity is also connected with an increased number of skin infections such as candidal intertrigo, furunculosis, erythrasma, tinea cruris, and folliculitis. It also predisposes to skin problem such as acne [90,91,92].

## 7. Psychological Consequences of Obesity

Contemporary culture obsessed with a slim figure, aversion to fat observed both in adults and children, and blaming the obese can be conducive to low self-esteem and negative self-image among individuals who do not match the stereotype of an attractive, healthy person. Thus, obesity often predisposes to depression; for instance, more or intensified symptoms of depression were recorded in people waiting for surgical treatment than in people with normal weight. It was also demonstrated that obesity was associated with a greater risk of identifying various depressive states throughout one’s life [93]. For example, studies have shown that mothers, women whose BMI exceeded 30, were five times more exposed to depression [93]. Obese people were found to have a higher level of fear, a negative image of their own bodies, and worse life quality [93]. Obese women were more dissatisfied with the image of their bodies than obese men [93]. People who developed obesity before the age of 16 reported dissatisfaction with their bodies and, in particular, with the waist and abdomen. Their self-evaluation is more negative than that of less obese people [93].

The awareness of the role and significance of nutrition in human life and knowledge of the needs of the body, as well as of the nutritional value of available products, are very important from the point of view of obesity. Taste preferences are also significant [94].

It was also noted that a relationship existed between obesity and the period of giving up smoking and. in previous times when military service was compulsory for young men, the termination of service [95].

From the psychological perspective, an obese person is an individual who perceives himself or herself as an obese person and feels obese. In the behavioral theory, incorrect behavior and eating habits acquired in childhood are considered to be a cause of obesity since the intake of energy by a person at the later stages of life is much higher than needed.

In psychoanalysis the reasons for obesity are sought for in the specific relations in the family of an obese child. Obesity is not a manifestation of constitutionally-determined developmental disorders of the child but a result of the relation between the father and mother and the parents and child. According to this approach, the obesity of the child is a consequence of failure of the emotional relations in the family. In the psychosomatic model obesity can be deemed a somatic reflection of failure in the functioning of the mental mechanisms of an individual. Obesity is an effect of inadequate interpretation of the child’s cry by its parents. Parents think that a child crying out of emotion actually cries because he or she is hungry. As a consequence the child in emotional discomfort looks for food to satisfy its needs [38].

In situations causing negative emotions, bad mood, and low spirits, food becomes a cure for various problems. Likewise, the urge for binge eating is connected with experiencing emotional rejection, anger, and hate. Overeating can be an expression of a protest, a reaction to feeling guilty, a justification of a life’s failure, and an expression of the lack of or insufficient satisfaction of the needs of an individual. Children who feel unloved or unaccepted by their parents, who experience emotional rejection, suppress their feelings and emotions by looking for comfort in food. Food helps them forget about the difficult things for a while. With time these beliefs are transferred to other areas of life. Empirical study confirms that emotion regulation, in the relation between parental rejection and emotional eating, impact on obese youngsters. The findings highlight the importance of assessing the emotional bond between mother and child and the emotion regulation of the youngster and their positive influence in the treatment of pediatric obesity [96].

An inability to cope with difficult situations and stress also contribute to overeating. Periods of intensified intellectual work, tension, and conflict make people consume excessive amounts of food [97,98]. Empirical studies also show that reducing strong emotions and stress reduces emotional eating when it comes to women with obesity [97]. 

From the point of view of society, obese people are stigmatized due to their weight. They are neglected and discriminated in everyday life. A person who is stigmatized due to excess weight is negatively stereotyped, which enhances mistreatment and discrimination. At present, even a “social stereotype of an obese person” exists. Generally, overweight people are not considered very attractive. They are also ascribed worse character traits, such as lack of strong will, self-control, and self-care, as well as worse professional qualifications. The term “stigma of obesity” is used with equal frequency. Additionally, attention must be paid to the fact that the main negative effect of stigmatization usually does not refer to the physical consequences of the specific trait but its psychological and social consequences. Most traits associated with capability lead to rejection and isolation and, as a result, have a negative effect on the psychosocial functioning of an individual. Obese people very often interpret their experiences with reference to obesity [98].

## 8. Prevention of Obesity

It is common knowledge that prevention is better than cure. It also refers to obesity; thus, it is so important that the society is made aware of factors causing obesity and of risks related to being obese [99]. 

All the measures aiming to prevent obesity should contain recommendations concerning a change in individual health behavior, cooperation with a physician, nutritionist or psychologist, as necessary, in order to control the method of nutrition, change eating habits, and increase physical activity [100]. Although lack of physical effort is not an immediate cause of obesity, it is certainly a factor fostering such a condition. High motivation and active participation of the person attempting to lose weight are also very significant and have a positive effect on changing the lifestyle and eliminating bad eating habits [3,100,101]. 

Physical effort facilitates maintaining energy balance. During light work the body burns 1.5–3 kcal/min and, during intensive exercise, 7–12 kcal/min. As a part of obesity treatment it is recommended to exercise for 20–60 min a day from 3–5 times a week. The exercise can be gymnastics, jogging, swimming, walking, or games such as badminton or volleyball [102]. Additional advantages related to increased physical activity include reduction of body fat, increase in the muscle and bone mass, improved fitness, reduced concentration of insulin and improved lipid profile, and reduced resting and stress blood pressure and heart rate [100]. Physical activity facilitates maintenance of a long-term diet regime, improves one’s mood and mental health, as well as emotional condition [103,104].

In preventing obesity eating full-value products such as wholemeal bread, lean cured meat and cheese, lean fish, lean meat and processed meat, boiled vegetables, e.g., potatoes, is recommended [105]. Adding herbs, for example, dill, parsley leaves, basil, oregano, and thyme, to food is also recommended. Recommended beverages include skimmed milk, non-carbonated mineral water, chicory coffee without sugar, and vegetable juice without sugar [106].

## 9. Summary

Obesity is one of the numerous diseases of civilization in the contemporary world. It is a risk factor and a direct cause of many severe diseases, not only somatic, but also mental. Obesity can also be a reason for social exclusion. In consequence, obesity can even lead to death. Not only adults, but also children suffer from obesity and their number has been growing year after year. This is the state of affairs throughout the world and in particular this problem touches Polish society.

The world has also changed stereotypes regarding obesity. An obese person is no longer a reflection of well-being, but rather of negligence. Before, many people associated obesity with an unattractive, unshapely figure only. Now, this view has changed—more and more people realize the risks associated with being obese and are aware of the negative consequences for their health. Therefore, it is necessary to undertake measures to make societies aware of how bad eating habits and lack of physical activity contribute to an increase in the incidence of obesity and, in consequence, to the development of severe diseases which are difficult and very costly to cure. Increasing social awareness and promoting physical activity have, thus, become the objectives of public health in the care for maintaining good health and creating a correct lifestyle based on the idea that prevention is better than cure. 
